# Post endodontic Aspergillosis in an immunocompetent individual

**DOI:** 10.4317/jced.52247

**Published:** 2015-10-01

**Authors:** Aadithya B. Urs, Hanspal Singh, Kalpana Nunia, Sujata Mohanty, Shalini Gupta

**Affiliations:** 1 MDS, Professor & Head, Department of Oral & Maxillofacial Pathology,Maulana Azad Institute of Dental Sciences, New Delhi, India, BSZ Marg, New Delhi-110002; 2 MDS, Senior Resident, Department of Oral & Maxillofacial Pathology, Maulana Azad Institute of Dental Sciences, New Delhi, India, BSZ Marg, New Delhi-110002; 3 BDS.(Post Graduate Student), Department of Oral & Maxillofacial Pathology, Maulana Azad Institute of Dental Sciences, New Delhi, BSZ Marg, New Delhi-110002; 4 Professor & HOD, Department of oral Surgery, Maulana Azad Institute of Dental Sciences, BSZ Marg, New Delhi-110002; 5 MDS (Mumbai), FDSRCS(Edin), FAAOM, Associate professor, Oral Medicine and radiology, Maulana Azad Institute of Dental Sciences, New Delhi, BSZ Marg, New Delhi-110002

## Abstract

Non-invasive aspergillosis in immunocompetent individuals subsequent to post endodontic treatment can involve the maxillary antrum. An early and accurate diagnosis will aid in prompt and effective treatment. A 35 year old female patient reported with a painful nasomaxillary swelling. Previous records revealed the failure of the endodontic treatment of maxillary left second premolar which was subsequently extracted. Root piece was accidently left behind which resulted in a painful nasomaxillary swelling. The extraction socket was curetted and tissue was sent for histopathological examination, which revealed abundant septate fungal hyphae with numerous spores characteristic of Aspergillus. The patient showed marked improvement in the symptoms with systemic itraconazole at 3 months follow up and complete resolution occurred within 6 months. Inclusion of aspergilloma infections in the differential diagnosis is advocated when patients present with post-endodontic nasomaxillary swelling.

** Key words:**Aspergillosis, fungal sinusitis, post root canal treatment.

## Introduction

Aspergillosis of the paranasal sinuses has been regarded as a rare disease. However, during the last 2 decades, an increase in the number of cases has been reported (1). Aspergillus species are ubiquitous fungi acquired by inhalation of airborne spores and thus usually involve the bronchopulmonary region. They may cause severe life-threatening infections, especially in immunocompromised hosts (1). Occurrence in immunocompetent individuals has rarely been reported. It has been suggested that root canal trea-ted teeth with overextension of the root canal sealer into the sinus might be the main etiological factor for aspergillosis of the maxillary sinus in healthy patient (2). The present case is a rare instance of sinonasal aspergillosis in an immunocompetent person. Its development secondary to root canal treatment and Oro-Antral-Fistula (OAF) adds to its rarity. This case highlights the importance of thorough examination and investigation in cases of post endodontic painful swelling, especially in the nasomaxillary region so that the complications arising due to inappropriate or delayed treatment in such patients can be minimized.

## Case Report

A 35 year old healthy female patient reported with pain in the upper left back tooth region for the past 8 months. On thorough examination of her dental records, it was found that pain initiated as a localized dull ache in the upper left second premolar due to dental decay. The patient visited a private dental practitioner and underwent root canal treatment in the offending tooth. Subsequently, there was no apparent improvement. So after a period of 2 months, the root canal treated tooth was extracted at the same clinic due to persistence of symptoms. During extraction, a small root piece was accidentally broken and pushed inside the maxillary sinus. After a week, extraction socket was unsuccessfully explored to remove the root piece.

**Figure 1:**
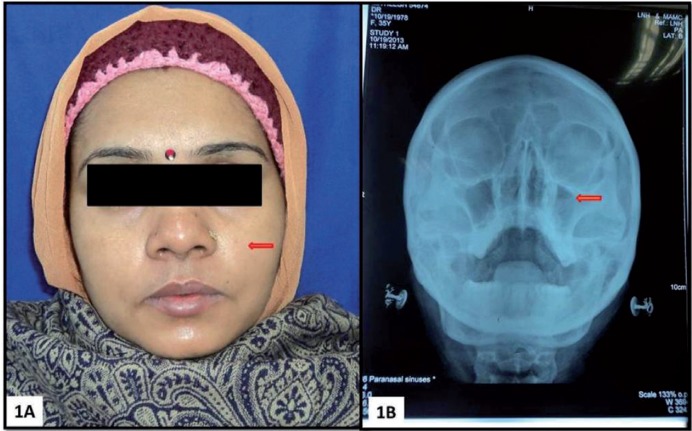
A) Extraoral view showing mild ill-defined diffuse swelling of the left nasomaxillary region. B) Paranasal sinus view showing haziness in lower two-third of the left maxillary sinus.

**Figure 2:**
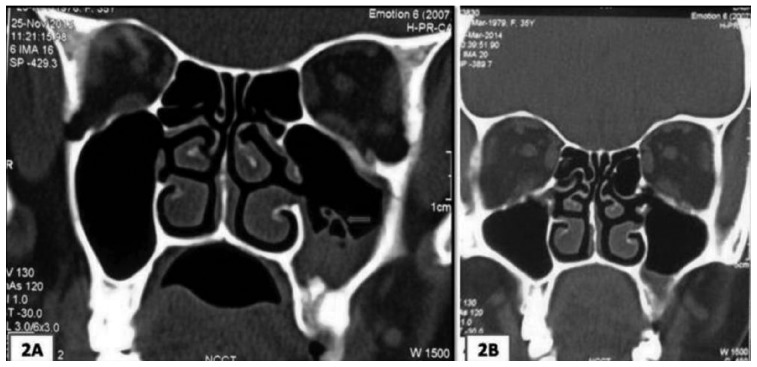
A) Contrast enhanced CT showing mucosal thickening in left maxillary sinus and radiopaque mass in the periapical region of 25. Irregular bony margins and discontinuity in the floor of the left maxillary sinus can also be noted. B) Post-operative CT scan showing marked reduction in mucosal thickening in the left maxillary sinus at 3-month follow up.

Subsequent to this, after a month, the patient reported to our institute with a diffuse swelling and the dull ache involving the left side of the face. The patient also complained of sore throat and difficulty in deglutition. Occasional nasal obstruction and unilateral headache involving the left side of the face were also reported. On extra oral examination, mildly ill-defined, diffuse, tender swelling was noted in the left nasomaxillary region (Fig. 1A). The skin overlying the swelling appeared normal. On intraoral examination, maxillary left second premolar was clinically missing and mucosa around its extraction socket, showed a small sinus opening. Maxillary left first premolar and first molar were found to be tender on percussion. No lymphadenopathy was noted. Paranasal Sinus (PNS) view showed haziness in the lower two-third of the maxillary sinus (Fig. 1B). Contrast enhanced Computed tomography (CECT) scan showed mucosal thickening in left maxillary sinus and radiopaque mass in the periapical region of the second premolar (Fig. 2A). Irregular bony margins and discontinuity in the floor of the left maxillary sinus were also noted which indicated the possibility of Oro-Antral-Fistula (OAF). Previous radiographs of root canal treatment could not be obtained from the patient. Chest radiograph did not show any abnormalities. Routine blood tests were also normal. The extraction socket was surgically explored. Curetted tissue associated with the fractured root piece was removed along with the infected sinus lining and submitted for histopathological diagnosis.

**Figure 3:**
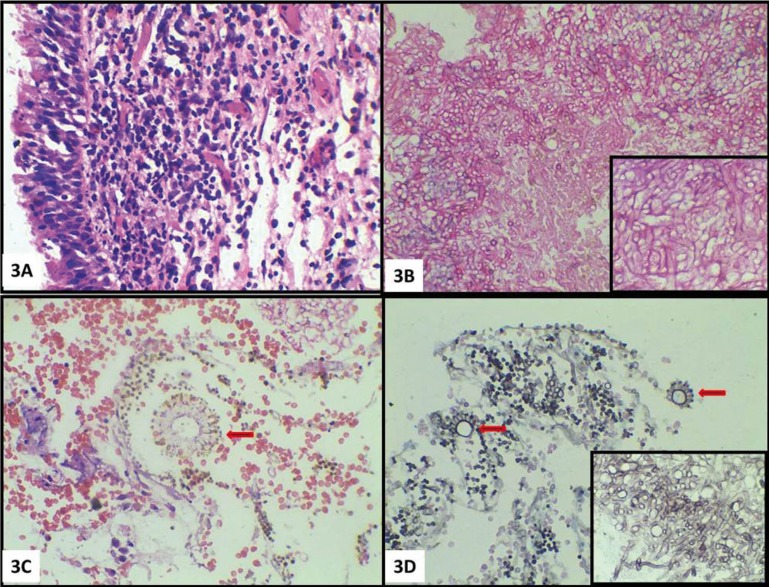
A)Pseudostratified ciliated columnar epithelium representing maxillary sinus lining with underlying connective tissue stroma showing dense infiltrate of chronic inflammatory cells (hematoxylin-eosin [HE], X 40). B) Abundant fungal hyphae with numerous spores (HE, X40). Inset shows septate fungal hyphae (HE, X100). C) Condidiophores with uniseratephialides surrounding the vesicle (HE, X40). D) Conidiophores along with elliptical to globose conidia (Grocott’s Methenamine Silver [GMS], X40). Inset shows septate hyphae with dichotomous branching at 45° (GMS, X100).

Hematoxylin and eosin stained sections revealed pseudostratified ciliated columnar epithelium representing maxillary sinus lining. Underneath the lining, the connective tissue stroma was densely infiltrated with chronic inflammatory cells and abundant dilated capillaries (Fig. 3A). Other sections of soft tissue studied showed connective tissue infiltration by abundant fungal hyphae with numerous spores. The section predominantly showed branched septate hyphae measuring 3 to 6 µm in diameter (Fig. 3B). Few conidiophores were also seen (Fig. 3C). Staining with Grocott’s Methenamine Silver (GMS) confirmed the presence of septate hyphae with dichotomous branching at 45. (The conidiophores were thick-walled, long and 10 to 20 µm in diameter below the vesicle. Vesicles were globose to subglobose, 10 to 65 µm in diameter and were surrounded by phialides. Phialides were uniserate with primary branches measuring up to 10 µm in length. The conidia were elliptical to globose and measured 3.5 to 4.5 µm in diameter (Fig. 3D). A diagnosis of Aspergillosis caused by *A. flavus* was made on histopathological diagnosis, since the characteristic fruiting bodies were evident in the sections studied.

Following the confirmation of histopathological diagnosis as Aspergillosis, the patient was advised 200 mg twice daily of systemic itraconazole. Also, the patient was subjected to further investigations, including High resolution computed tomography (HRCT) of the chest to rule out bronchopulmonary involvement. All reports were found to be negative and the patient was confirmed as immunocompetent. At 3-month follow-up, the symptoms had completely subsided. CECT showed marked reduction in mucosal thickening (Fig. 2B). Complete closure of oro-antral communication and healing of extraction socket was noted at 6 months follow up, subsequent to which antifungal drug was stopped. Currently the patient is disease free and apparently healthy.

## Discussion

Aspergillosis of head and neck region, primarily affects the nasal cavity and paranasal sinuses. *Aspergillus* as a pathogen cannot actively penetrate undamaged and intact mucus membrane or skin as it lacks keratolytic enzymes. The most important human pathogenic *Aspergillus* are *A. fumigatus, A. circumdatus, A. flavus* and *A. niger*, amongst which *Aspergillus flavus* involves predominantly the paranasal sinuses followed by other species (3).

Sinus mycoses can be classified as: non-invasive, invasive and allergic variants. The non-invasive form called *Aspergillus mycetoma*, aspergilloma, or fungus ball occurs mostly in healthy individuals. Usually only one sinus, especially the maxillary antrum, is affected symptomatically or asymptomatically (4). Radiographically, the unique appearance of a densely opaque foreign body reaction in the maxillary sinus is considered as a characteristic finding. These objects called foreign bodies, concrements, or antroliths are usually in the center or near the orifice of the maxillary sinus (5).

Beck-Mannagetta *et al.* demonstrated that maxillary sinus aspergillosis is in most cases caused by excess root-filling materials that contain zinc oxide and formaldehyde (2). Present case showed features of chronic sinusitis and had a history of root canal treatment as well as surgical intervention to remove the broken root piece. So, both the intrusion of root-canal filling material as well as surgical exploration of an extraction socket under septic conditions could be the probable cause of aspergilloma in the present case. But, since the symptoms aggravated only after the surgical exploration of extraction socket, fungal infection secondary to septic surgical intervention is more likely. The clinical and radiographic findings were compatible with a diagnosis of non-invasive aspergillosis.

Khongkhunthian *et al.* reported two cases of young healthy female patients with clinical and radiographic findings similar to the present case. Surgical inspection of both the patients revealed aspergillomas along with overextended root canal cement in the maxillary sinus subsequent to endodontic treatment of maxillary first molars. Eugenol gradually loses its fungicidal and fungistatic property and leaves the zinc oxide, which favours growth of *Aspergillus species* (4). However, in the present case, a persistent OAF and broken root piece were observed.

Similarly, Giardino *et al.* reported a case of healthy male with overextension of root canal sealer in the maxillary sinus (6). This could produce inflammatory reaction and create an environment for *Aspergillus* growth. Zinc influences not only the metabolism of *Aspergillus* but also mucosal hyperemia and mucociliary paralysis associated with potential epithelial dysfunction. This results in deposition of calcium phosphate, which constitute a nidus for spores (7). Microscopic examination showed numerous branching hyphae characteristic of *Aspergillus* (6).

Aspergillosis of maxillary sinus secondary to foreign body reaction to amalgam has also been reported. Burnham et al reported aspergillosis in a 46 year old male patient, post extraction of a heavily restored and infected upper right second molar. CT showed considerable mucosal thickening arising from the antral floor with an irregular focus of hyperdense material. Histopathological examination of the surrounding tissue showed large collection of *Aspergillus* (8).

Though classically hyphae of *Aspergillus* are described as acute branching septate hyphae, sometimes it becomes difficult to differentiate them from hyphal forms of other fungi such as *Pseudallescheri aboydii*, the *Fusarium* Spp. and occasionally with the *Candida* spp. Thus, a microbiological isolation by culture is often required for confirmation, which, considering the ubiquitous nature of *Aspergillus* is difficult to get. However, when the fruiting bodies of *Aspergillus* are seen, diagnosis can be considered and even species sub typing can be attempted at the histopathology level (9). Blood cultures and serological testing may not be always reliable in establishing the diagnosis and cultures may be negative even in the presence of the typical histological feature of Aspergillosis (5). Most species of Aspergillus are susceptible to cycloheximide present in the culture media. Cycloheximide is a common component of culture media, added to inhibit the growth of rapidly growing contaminating molds. Hence false negative results may be encountered if a culture is performed on the cycloheximide containing media. Recent advances in diagnosing Aspergillosis include Real-Time PCR, Galactomannan antigen detection and estimation of aflatoxin by enzyme linked immunosorbent assay (ELISA) (10). In the current case, histopathological examination proved to be precise with characteristic fruiting bodies. Hence, further diagnostic investigations were not carried out.

Histopathologically, the conidiophores of *A. flavus* are thick walled and produce phialides which cover almost the entire area around the central large vesicle. On the other hand, conidiophores in *A. fumigatus* form expanded flask-shaped vesicle. The phialides bend upward, paralleling the axis of the conidiophores. Hence this differentiating feature between *A. fumigatus* and *A. flavus* was used to identify the species on the histopathological section in the current case, which was a unique feature observed (11). Identifying *Aspergillus* species may have diagnostic value, as certain species are associated with higher mortality and increased virulence and vary in their resistance to antifungal therapy (12).

Local antimycotic agents such as itraconazole and voriconazole, are used for its treatment, depending upon the severity of infection (13). Recently, the use of Voriconazole in the primary treatment of invasive aspergillosis has been recommended due to its better tolerance and less toxicity (14). Complete surgical debridement and curettage are advocated prior to antimycotic therapy.

## Conclusions

Based on the clinical, radiographic, CT examination and histological findings, this case was diagnosed as non-invasive aspergillosis secondary to unsuccessful extraction and development of oro-antral fistula. Aspergillosis can develop even in the immuno-competent patients and should be kept as a differential diagnosis when a patient presents with a painful nasomaxillary swelling post endodontic treatment or post extraction. Careful examination and accurate diagnosis of such patients help in prompt treatment, thus minimizing cost, toxicity and other complications due to inappropriate treatment strategy.
